# Optimization of a molecularly defined tuberculin formulation: recombinant fusion proteins and epitope surgery

**DOI:** 10.1128/jcm.00552-25

**Published:** 2025-08-29

**Authors:** Sonya Middleton, Mahavir Singh, Michael Coad, Si Palmer, Tom Holder, Sabine Steinbach, Rebecca Hardiman, H. Martin Vordermeier, Gareth J. Jones

**Affiliations:** 1 Department of Bacteriology, Animal and Plant Health Agency 16232, Addlestone, United Kingdom; 2 Lionex Diagnostics and Therapeutics GmH, Braunschweig, Germany; University of California, Davis, Davis, California, USA

**Keywords:** MDT, molecularly defined tuberculin, skin testing, tuberculin, bovine tuberculosis, purified protein derivatives

## Abstract

Bovine tuberculosis is an infectious livestock disease of global economic and zoonotic importance. Surveillance programs are largely dependent on the skin test that utilizes purified protein derivatives (PPDs) of tuberculin. PPDs suffer from a number of limitations regarding their production, characterization, and performance. Recently, we developed a molecularly defined tuberculin (MDT) that overcame these limitations. The MDT formulation comprises eight antigens, initially presented as seven recombinant proteins (ESAT6, CFP10, Rv3615c, Rv3020c, Rv1789, Rv3478, and Rv3810) and one 20-synthetic peptide pool representing Rv3616c; the recombinant Rv3616c could not be produced. In this paper, we describe the steps taken to simplify the MDT formulation. First, seven of the eight proteins were formulated as three recombinant fusion proteins. Second, the non-immunogenic regions of Rv3616c were identified, using overlapping peptides to probe T-cells from infected cattle. A novel variant, Rv3616c-S4, was designed and then formulated (i) as a stand-alone protein in the MDT-S4 formulation and (ii) fused to Rv1789 for use in the MDT-F formulation. These novel MDT formulations gave comparable signal strength to PPD-B in experimentally infected animals. The MDT-F, comprising only fusion proteins, performed as well as the comparative cervical tuberculin skin test, in terms of relative sensitivity (>2 mm; 96%, 95% CI 80–100, *n* = 24) and specificity (>2 mm; 100%, 95% CI 89%–100%, *n* = 30). In conclusion, we have developed a novel, simplified MDT formulation that has significantly advanced our efforts toward licensure and which has the potential to replace the PPDs developed in the 1930s.

**IMPORTANCE:**

Bovine tuberculosis is an infectious livestock disease of global economic and zoonotic importance. Surveillance programs are largely dependent on the skin test, which utilizes tuberculins. These are crude protein extracts of live bacterial cultures, which suffer from a number of limitations regarding their characterization, standardization, production, and performance. We aim to develop a skin test reagent composed of eight defined antigens that could replace the tuberculins. This study describes the refinement of this “molecularly defined tuberculin” (MDT) reagent into a fusion protein formulation comprising all eight antigens. The MDT is well defined, easily standardized, and delivers good test performance in experimentally infected, non-infected cattle and cattle sensitized to Johne’s disease. The fusion protein formulation is an important step on the developmental pathway to a registered and marketable product.

## INTRODUCTION

Bovine tuberculosis (BTB), caused by members of the *Mycobacterium tuberculosis* complex such as *Mycobacterium bovis*, *Mycobacterium caprae*, and *Mycobacterium orygis*, is an infectious and zoonotic disease of cattle with a broad host range and a significant global economic, animal welfare, and human health impact (1). While many high-income countries have successfully eradicated, or are close to eradicating, the disease (2–4), BTB continues to exact a significant toll on the farming sector in many low- and middle-income countries and to hinder eradication efforts in countries which have a wildlife reservoir, such as the European badger (*Meles meles*) in the UK and Ireland (5, 6). Globally, it is estimated that BTB has an annual cost, through losses in productivity and the cost of control programs, of approximately US$3 billion (7); in the UK alone, the combined costs of the eradication effort and the costs to the cattle industry surpass £150 million per year (8).

In many countries, surveillance and eradication programs for BTB in cattle and other livestock are primarily reliant on the application of ante-mortem tuberculin skin testing. The antigens used for such skin tests, and the supplementary UK/EU-approved interferon-gamma (IFN-γ) blood test, are purified protein derivatives (PPDs) of tuberculin, which were originally developed in the 1930s. The most basic form of tuberculin skin testing utilizes a PPD prepared from *M. bovis* (PPD-B; single cervical tuberculin test or caudal fold test). To increase test specificity, an additional injection of avian PPD (PPD-A) prepared from *Mycobacterium avium subsp. avium* is used alongside PPD-B, in the comparative cervical tuberculin test (CCT). In the CCT, only animals that develop a larger reaction to PPD-B compared to PPD-A are removed, with different cut-offs being applied according to the epidemiological context.

The successes of the numerous control and eradication programs reliant on PPDs cannot be overlooked; however, these reagents suffer from a number of limitations, from ease of manufacture to standardization and cross-reactivity. Both PPDs are crude protein extracts of live bacterial cultures, and as such, the biorisk inherent in the manufacture of PPD-B mandates bio-containment level 3 (BCL3) production facilities. Furthermore, the extracts’ active antigenic components are not well defined and therefore poorly standardized, and this in turn impacts test accuracy, an issue further compounded by any differences in production methods between manufacturers (9). PPD potency, an important factor in the performance of the skin test, can also vary (10) and is determined using the *M. bovis* infection guinea pig potency assay. This assay is also dependent on BCL3 animal accommodation facilities, and being an *in vivo* bioassay, it is inherently variable and difficult to standardize. Furthermore, the potency assessments themselves require validation against an aged international standard in limited supply (11). Due to PPD cross-reactivity with other mycobacteria, the performance of the skin test can be compromised by host exposure to some environmental mycobacterial species. The inclusion of PPD-A in the CCT improves specificity, but this comes at the expense of the sensitivity of the test (12). PPD performance is also compromised by infection with, or vaccination against, Johne’s Disease, caused by *M. a. subsp. paratuberculosis* (12).

In order to overcome these limitations, as per the recommendation of the Bovine TB Strategy Review (13), we recently developed a molecularly defined tuberculin (MDT) formulation consisting of eight specific antigens (ESAT6, CFP10, Rv3615c, Rv3020c, Rv1789, Rv3478, Rv3616c, and Rv3810), selected to boost signal strength and complementation in the skin testing of cattle not vaccinated with Bacillus Calmette–Guérin (BCG), i.e., the MDT was not designed to differentiate infected from vaccinated animals (14). The original MDT formulation represented seven of these eight antigens as single recombinant proteins (Table 1). Rv3616c, which could not be formulated as a recombinant protein as it lysed its *Escherichia coli* expression host, was presented as a pool of 20 synthetic peptides. In the present study, we sought to (i) reduce the complexity of the MDT formulation by combining seven of the eight antigens in recombinant fusion proteins, (ii) design and produce a novel Rv3616c variant to overcome the production issues, and (iii) produce a two-antigen fusion protein utilizing the new Rv3616c variant, which would enable us to make an MDT fusion formulation of minimal complexity. The novel Rv3616c variant was designed by mapping Rv3616c’s dominant antigenic regions, using T-cells from *M. bovis*-infected cattle. Our hypothesis was that deleting any non-antigenic regions from the wild-type sequence would disrupt Rv3616c’s tertiary structure, thereby rendering it non-lytic to the *E. coli* cells. In this paper, we describe the successful outcome of these approaches, leading to a refined MDT formulation of minimal complexity that could be taken forward for further development into a marketable product.

**TABLE 1:** Summary of the MDT formulation compositions^*a*^^,^^*b*^

Formulation	Composition
MDT	Rv3615c, ESAT-6, CFP-10, Rv3020c, Rv1789, Rv3810, Rv3478 Rv3616c peptide pool (*n* = 20)
MDT-F20/22/13	Rv3615c_ESAT-6_CFP-10_Rv3020c Rv1789_Rv3810 Rv3478_Rv3810 Rv3616c peptide pool (*n* = 20, 22, or 13)
MDT-S4	Rv3615c_ESAT-6_CFP-10_Rv3020c Rv1789_Rv3810 Rv3478_Rv3810 Rv3616c-S4
MDT-F13.1	Rv3615c_ESAT-6_CFP-10_Rv3020c Rv1789 Rv3478_Rv3810 Rv3616c peptide pool (*n* = 13)
MDT-F	Rv3615c_ESAT-6_CFP-10_Rv3020c Rv1789_Rv3616c-S4 Rv3478_Rv3810

^*a*^ Individual proteins are separated by ' , ' and fusion proteins are indicated by '_ '.

^*b*^ Evolution of the MDT formulations from individual proteins and peptide pools to an all-fusion protein product in MDT-F. Following the introduction of the tetra-fusion protein in MDT-F20, the formulations were adapted according to how Rv3616c was presented - from peptide pools of different sizes, to a stand-alone recombinant protein (S4), and finally as a fusion protein in MDT-F.

## RESULTS

### Simplification of the MDT formulation

Fusion proteins combining all eight of the MDT proteins were designed, of which three could be manufactured: (i) ESAT6, CFP10, Rv3615c and Rv3020c; (ii) Rv1789 and Rv3810; and (iii) Rv3478 and Rv3810 (Table 1). Unfortunately, none of the fusion proteins containing Rv3616c could be successfully expressed and purified. Therefore, Rv3616c was still presented as 20 overlapping synthetic peptides, which, together with the three fusion proteins, created a new formulation, MDT-F20 (Table 1).

**Fig 1:**
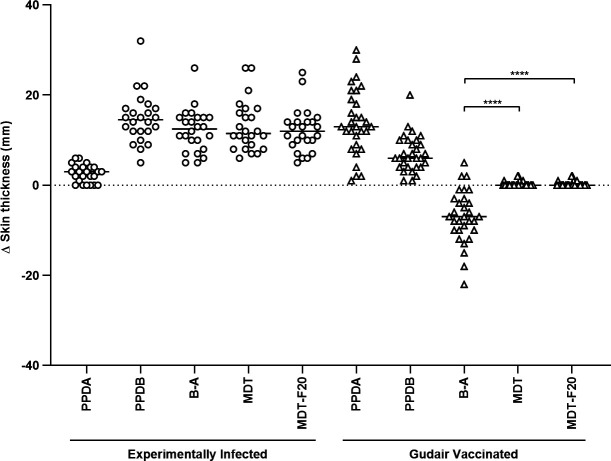
Comparison of MDT-induced skin test reactions in cattle. Calves experimentally infected with *M. bovis* (*n* = 24) or vaccinated with the Gudair vaccine (*n* = 30) were skin tested with PPDs, MDT, and MDT-F20 formulations 8 weeks post-infection or vaccination, respectively. Results are expressed as the difference in skin thickness between the pre- and post-skin test readings. Each symbol represents an individual animal, while horizontal lines represent group medians. **** *P* <0.0001, Friedman test with Dunn’s multiple comparisons test.

MDT and MDT-F20, alongside PPD-A and PPD-B, were then used to skin test 24 *M*. *bovis*-infected calves and 30 calves vaccinated against Johne’s Disease with the Gudair vaccine (Virbac Ltd, UK), to evaluate the sensitivity and specificity of the two MDT formulations, respectively (skin testing round 1). In Fig. 1, MDT and MDT-F20 presented with comparable skin test reactions in both experimental groups, and there was no significant difference between either of the MDT formulations and the B-A (CCT) reaction in the experimentally infected animals (Fig. 1; *P* > 0.05, Friedman test with Dunn’s multiple comparison test). When different cut-off values for positivity were applied (increase in skin thickness of >2, >3, and >4 mm), both MDT and MDT-F20 demonstrated 100% relative sensitivity (95% CI 86%–100%, *n* = 24). Furthermore, no test positive outcomes to either MDT or MDT-F20 were observed in the Gudair vaccinated animals, highlighting their high degree of specificity (100% relative specificity at all cut-offs; 95% CI 89%–100%, *n* = 30) in the face of strong sensitization to *M. a. ssp paratuberculosis* antigens (as evidenced by the strongly PPD-A biased CCT response in Fig. 1; *P* < 0.0001, Friedman test with Dunn’s multiple comparison test). Blood samples from both experimental groups were stimulated for the IFN-γ release assay (IGRA), as this is a valuable ancillary test applied alongside skin testing. The results (Fig. S1) confirmed the utility of MDT and MDT-F20 as sensitive and specific antigens for the IGRA, and the formulations did not differ in signal strength, while both were significantly stronger than the B-A *in vitro* (Fig. S1, experimentally infected animals, *P* > 0.05 and *P* < 0.0001, respectively, Friedman test with Dunn’s multiple comparison test).

### Generation of an Rv3616c variant that was not lytic to *E. coli* expression host cells

**Fig 2:**
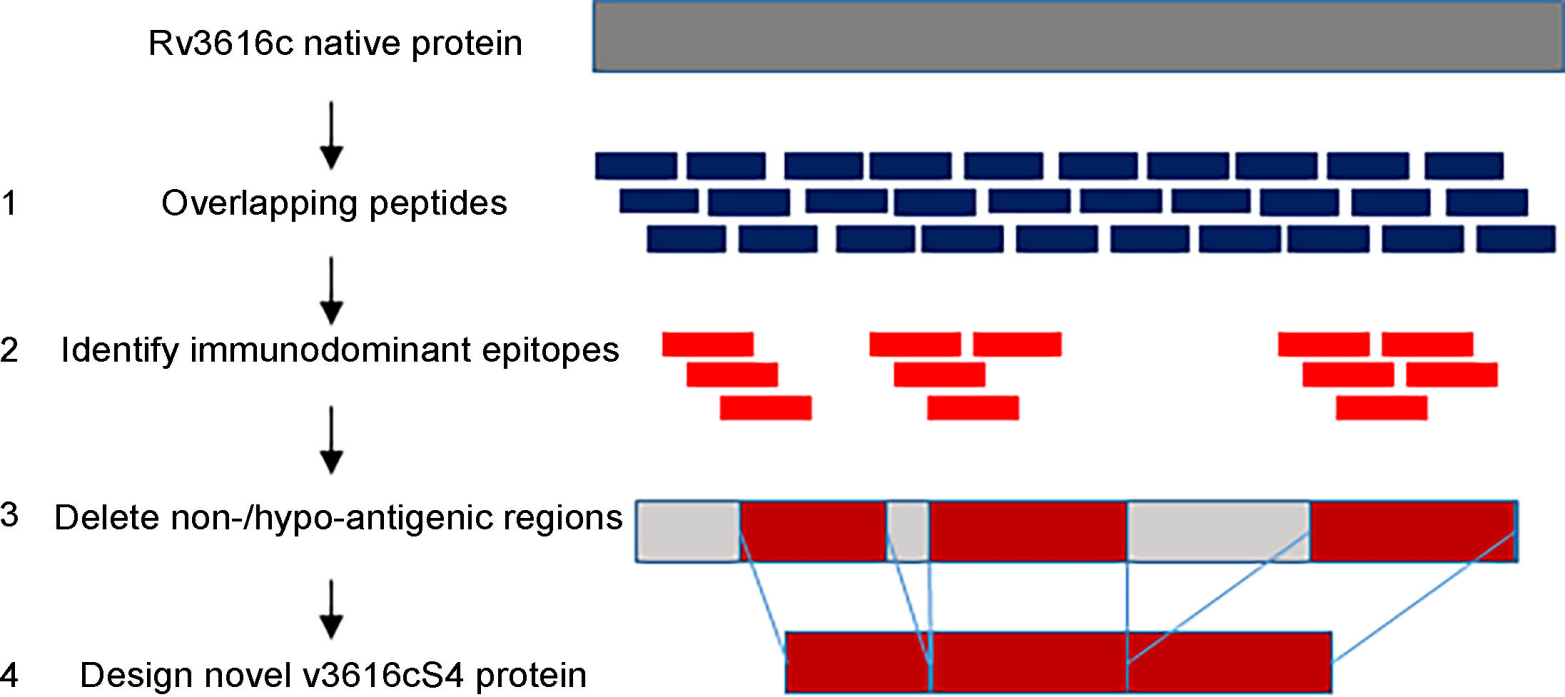
A schematic representation of the approach taken to design the novel recombinant Rv3616c-S4 protein. The epitope mapping of the wild-type sequence using 48 overlapping peptides (1) enabled us to distinguish between the immunodominant epitopes (2) and the non- or hypo-antigenic regions. As a result, 50 of the 392 amino acids were removed across three regions (gray sections in *3*).

**Fig 3:**
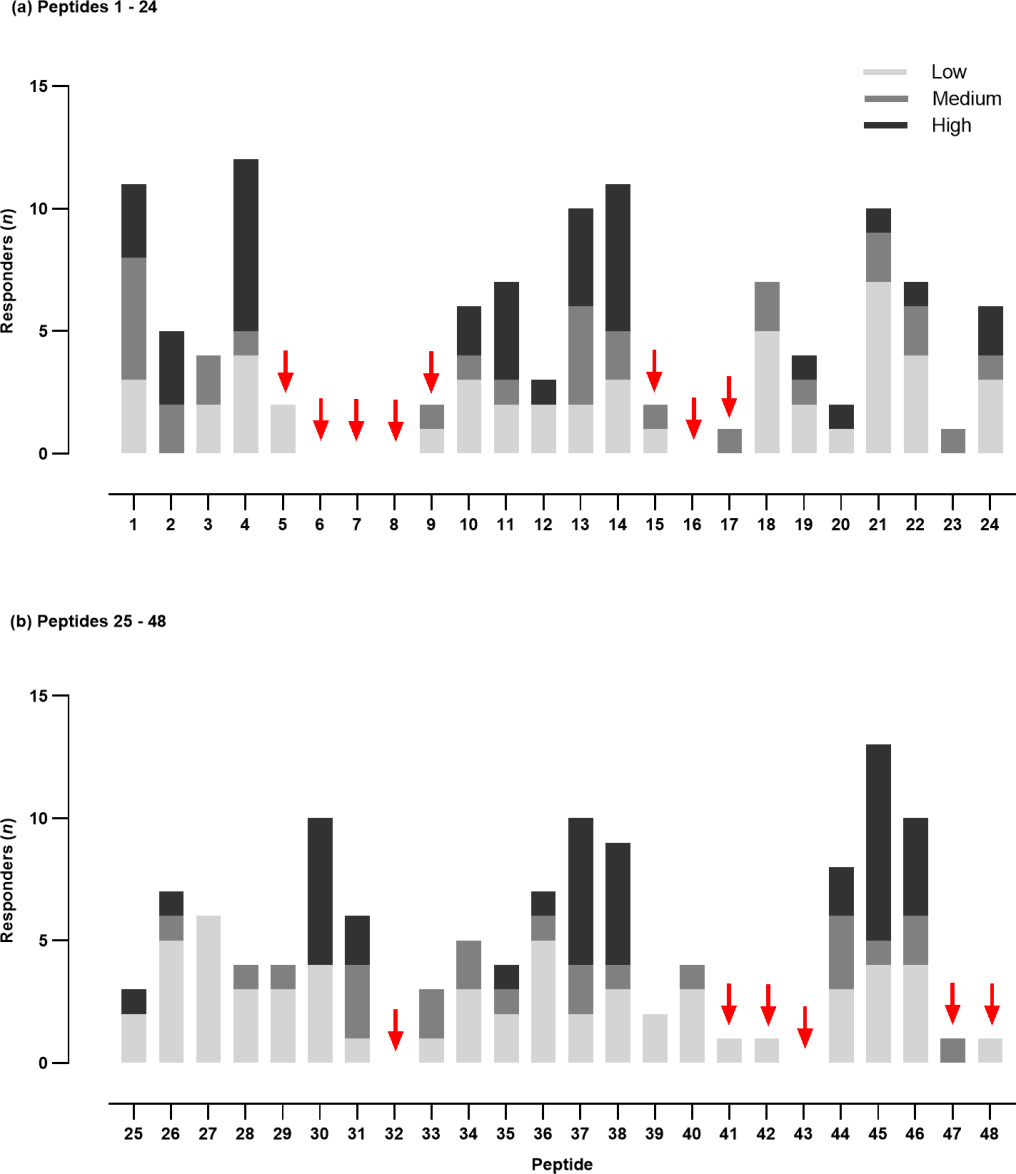
Mapping of T-cell epitopes within Rv3616c. PBMCs from *M. bovis*-infected cattle (*n* = 22) were stimulated for 20–24 h with a set of 48 overlapping synthetic peptides covering the amino acid sequence of Rv3616c, and the number of IFN-γ-producing cells enumerated using the IFN-γ ELISpot assay. The results are shown as the number of animals recognizing a particular peptide (*n* responders) with low, medium, or high strength based on the actual number of IFN-γ producing cells. Red arrows (*n* = 14) indicate epitopes which were not, or only infrequently, recognized for (**a**) peptides 1–24 and (**b**) for peptides 25–48.

**Fig 4:**
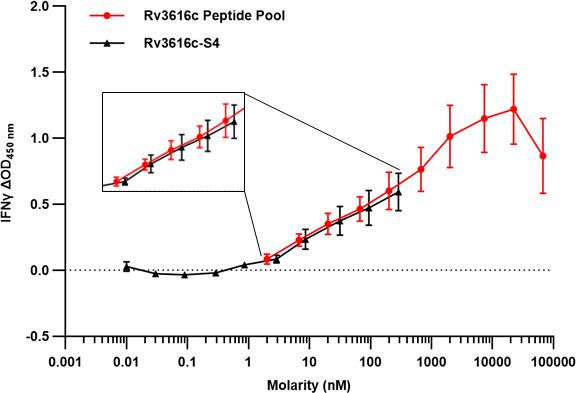
Evaluation of the novel Rv3616c-S4 protein *in vitro*. IFN-γ production as induced by the 22-peptide pool of Rv3616c and the Rv3616c-S4 variant. PBMCs from experimentally (*n* = 5) and naturally *M. bovis*-infected cattle (*n* = 5) were stimulated with antigens at 10 concentrations between 10 µg/mL and 0.0003 µg/mL, and IFN-γ production was measured by enzyme-linked immunosorbent assay. The dose-response curves were plotted using molarity (nM), and the inset panel shows the five molarities for which the responses of the protein and the peptide pool were compared. Each symbol represents a mean, and the error bars are SEM.

The aim was to identify and remove regions of the wild-type Rv3616c sequence that harbored no, or only weakly and infrequently recognized, epitopes capable of stimulating bovine T cells from infected animals. It was hoped that the disruption to the protein’s structure would render it non-lytic to the host cells, enabling the expression and purification of a novel recombinant protein (Fig. 2). A set of 48 overlapping synthetic peptides (20 amino acids long with an offset of eight residues; Table S2) was used in IFN-γ ELISpot assays with peripheral blood mononuclear cells (PBMCs) isolated from infected cattle. The non- or hypo-antigenic regions were readily identified based on the strength and frequency of the responses induced (Fig. 3; *n* = 14). Following the deletion of 50 out of 392 amino acid residues (highlighted in gray, Fig. S2), the novel variant, designated Rv3616c-S4, was purified using an *E. coli* expression system. Prior to skin testing, the immunogenicity of Rv3616c-S4 was evaluated *in vitro*, using the Rv3616c 22 peptide pool (Table S1) as a comparator and PBMCs from infected cattle. Figure 4 shows the IFN-γ dose-response curves plotted as ΔOD_450_ vs molarity (nM). Given that the novel S4 variant was demonstrably immunogenic, it was taken forward for evaluation *in vivo* in the skin test; there were no significant differences found between the IFN-γ responses of the protein and peptides at five comparable molarities (*P* > 0.05, Wilcoxon signed rank test; inset panel, Fig. 4).

### Performance of MDT formulations containing either Rv3616c-S4 or a reduced Rv3616c peptide pool

The next experiment (skin testing round 2) utilized the MDT-F22 formulation as a comparator for (i) the MDT-S4 containing the variant protein Rv3616c-S4 and (ii) MDT-F13, a formulation containing a smaller Rv3616c peptide pool (Table 1). To keep the formulation containing the Rv3616c peptide pool as an option, should alternative presentations of Rv3616c fail, additional mapping work was undertaken (M. Vordermeier and S. Middleton, unpublished data), and 9/22 poorly recognized or non-immunogenic peptides were removed (Table S1). The seven other proteins were represented in these MDT formulations by the same three fusion proteins (Table 1).

**Fig 5:**
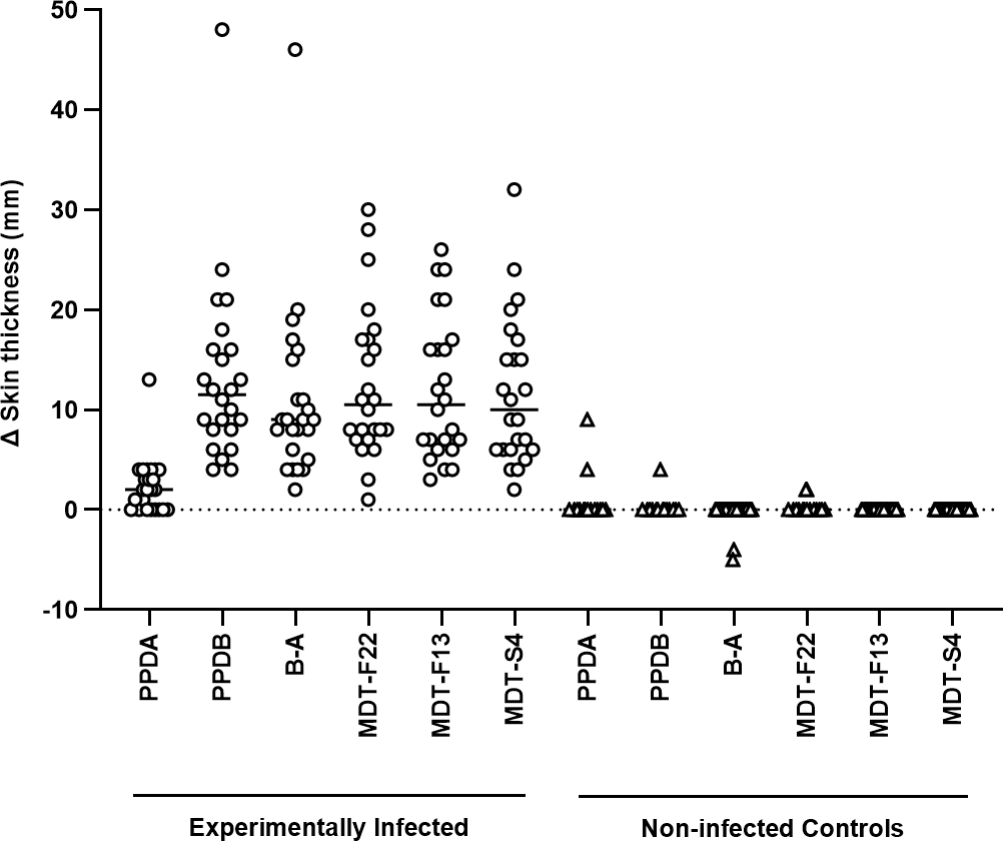
Comparison of MDT-F22, MDT-F13, and MDT-S4 induced skin test reactions in cattle. Calves experimentally infected with *M. bovis* (*n* = 24 calves from a vaccine/challenge experiment skin tested 12 weeks post-infection) and non-infected control animals (*n* = 30) were skin tested with PPDs and three MDT formulations (MDT-F22, MDT-F13, and MDT-S4). Results are expressed as the difference in skin thickness between the pre- and post-skin test readings. Each symbol represents an individual animal, while horizontal lines represent group medians.

These formulations were assessed for their ability to induce skin test responses in 24 calves experimentally infected with *M. bovis* (Fig. 5). To this end, we used animals from an ongoing vaccine efficacy study (see methods for *in vivo* skin testing round 2). As skin test responses did not differ significantly between the vaccinated and non-vaccinated groups (data not shown), all 24 animals were used to compare the different MDT formulations. All MDT formulations induced responses comparable to those of the B-A (CCT) and to PPD-B alone (*P* > 0.05, Friedman test with Dunn’s multiple comparison test); notably, both MDT-F13 and MDT-S4 were able to induce comparable reaction sizes to MDT-F22 (*P* > 0.05, Friedman test with Dunn’s multiple comparison test). Skin test data from the uninfected cattle also confirmed a high degree of specificity for both MDT-F13 and -S4 formulations (Fig. 5, no reactions >0 mm). Lastly, the IGRA was applied to supernatants from MDT-stimulated blood samples from both experimental groups. Although MDT-S4 displayed a slightly lower signal strength compared to MDT-F22 in infected cattle (Fig. S3, *P* < 0.0001, Friedman test with Dunn’s multiple comparison test), the responses were comparable to the B-A readout (*P* > 0.05, Friedman test with Dunn’s multiple comparison test) and all animals remained test positive (>0.1 ΔOD_450_). In infected cattle, there was no significant difference in signal strength between MDT-F22 and MDT-F13 (*P* > 0.05, Friedman test with Dunn’s multiple comparison test), demonstrating that *in vitro*, the reduction of the peptide pool did not negatively affect the formulation’s performance. Additionally, neither MDT-F13 nor MDT-S4 induced any positive responses in blood samples from non-infected animals, confirming their suitability as antigens for use in the IGRA.

### Evaluation of an Rv3616c fusion protein in the MDT-F formulation

While MDT-S4 and -F13 represented improvements in terms of simplification of the formulations, additional steps were taken to further rationalize the MDT into a minimal fusion product (Table 1). This involved (i) removing the duplicated Rv3810 from one of the fusion proteins and (ii) fusing the novel Rv3616c-S4 to one of Rv3478 or Rv1789. *In vitro* testing indicated no significant difference in the IFN-γ responses between formulations containing one or two Rv3810 proteins (S. Middleton and M. Vordermeier unpublished data), which paved the way for Lionex (Braunschweig, Germany) to attempt to manufacture Rv3616c fusion proteins with either Rv1789 or Rv3478, in different orientations. IGRAs following *in vitro* stimulations with PBMCs from infected cattle demonstrated that, of the two proteins produced, Rv1789_Rv3616c-S4 was significantly more immunogenic compared to Rv3616c-S4_Rv3478 (S. Middleton and M. Vordermeier unpublished data), and therefore, the former was selected for evaluation in the MDT-F formulation (Table 1).

**Fig 6:**
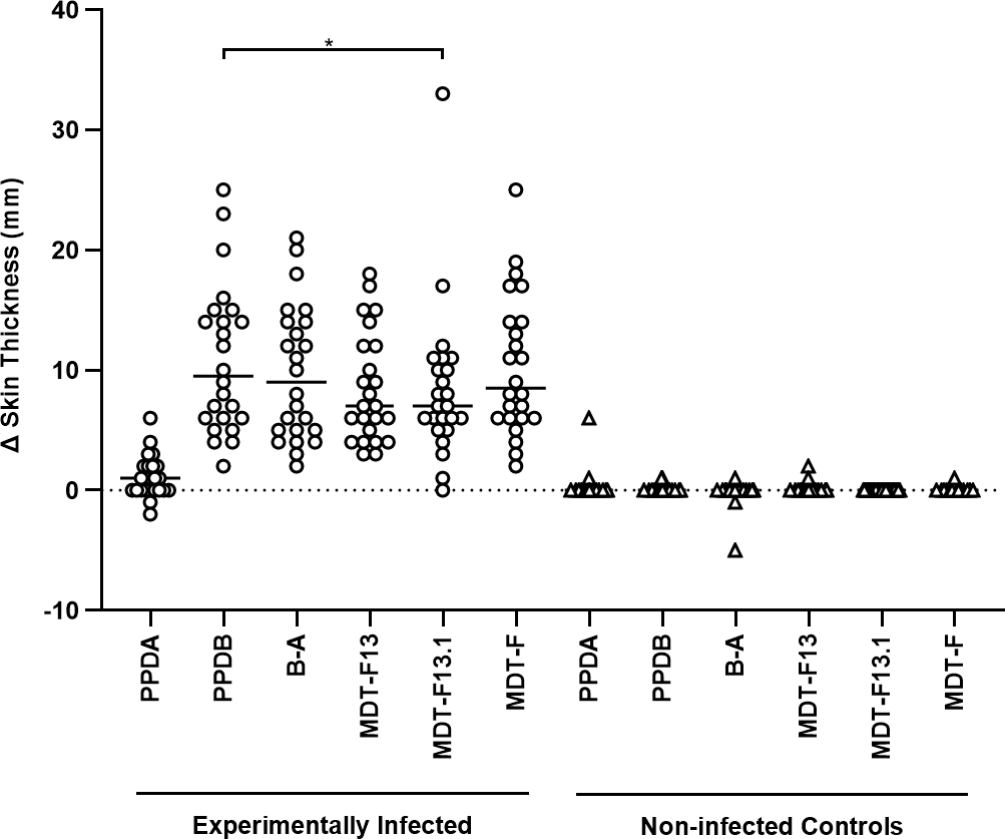
Comparison of MDT-induced skin test reactions in cattle. Calves experimentally infected with *M. bovis* (*n* = 24; skin tested 8 weeks post-infection) and non-infected controls (*n* = 30) were skin tested with PPDs, MDT-F13, MDT-F13.1, and MDT-F formulations. Results are expressed as the difference in skin thickness between the pre- and post-skin test readings. Each symbol represents an individual animal, while horizontal lines represent group medians. * *P* = 0.030, Friedman test with Dunn’s multiple comparisons test.

Skin tests (Fig. 6; skin testing round 3) in experimentally infected (*n* = 24) and non-infected control cattle (*n* = 30) evaluated three MDT formulations: MDT-F13 as the comparator, MDT-F13.1, and MDT-F (Tables 1 and S1). MDT-F13.1 was included to evidence whether the replacement of Rv1789_Rv3810 with Rv1789 would affect the formulation’s performance *in vivo*. Compared to MDT-F13, no significant differences in the magnitude of the skin test response were observed for either MDT-F13.1 or MDT-F, when tested in infected animals (*P* > 0.05, Friedman test with Dunn’s multiple comparison test; Fig. 6). Compared to PPD-B, significantly lower skin test reaction sizes were seen with the MDT-F13.1 formulation, but no significant difference was observed for MDT-F (*P* = 0.030 and *P* > 0.05, respectively, Friedman test with Dunn’s multiple comparison test; Fig. 6). In non-infected animals, no detectable increases in skin thickness were observed for MDT-F13.1 and only a small increase of 1 mm in one animal for MDT-F; two different animals showed small detectable increases in skin thickness of 1–2 mm to the MDT-F13 formulation.

A range of cut-off values was applied to the skin test data to estimate the relative specificity and sensitivity of the different MDT formulations and their performance compared to the corresponding values obtained for the PPDs (Table 2). In terms of relative specificity, at a cut-off of >2 mm, there was no difference between the MDTs, and they all performed as well as the CCT. Comparing relative sensitivity, the MDT-13 only performed better than MDT-F at one cutoff (>2 mm); the MDT-F performed as well or better at all other (more stringent) cutoffs. MDT-F13.1 detected fewer infected animals than the MDT-F at all cutoffs, while the latter performed as well as the CCT at >2 mm and better at >4 mm.

**TABLE 2:** Comparison of the MDT and PPD skin test performance at defined cut-off values.

Cut-off	% Specificity [95% CI] (*n* positive/total)			% Sensitivity [95% CI] (*n* positive/total)		
	MDT-F13	MDT-F13.1	MDT-F	MDT-F13	MDT-F13.1	MDT-F
>0 mm	93 [79, 99] (2/30)	100 [89, 100] (0/30)	97 [83, 100] (1/30)	100 [86, 100] (24/24)	96 [80, 100] (23/24)	100 [86, 100] (24/24)
>1 mm	97 [83, 100] (1/30)	100 [89, 100] (0/30)	100 [89, 100] (0/30)	100 [86, 100] (24/24)	92 [74, 99] (22/24)	100 [86, 100] (24/24)
>2 mm	100 [89, 100] (0/30)	100 [89, 100] (0/30)	100 [89, 100] (0/30)	100 [86, 100] (24/24)	92 [74, 99] (22/24)	96 [80, 100] (23/24)
>3 mm	100 [89, 100] (0/30)	100 [89, 100] (0/30)	100 [89, 100] (0/30)	92 [74, 99] (22/24)	88 [69, 96] (21/24)	92 [74, 99] (22/24)
>4 mm	100 [89, 100] (0/30)	100 [89, 100] (0/30)	100 [89, 100] (0/30)	75 [55, 88] (18/24)	83 [64, 93] (20/24)	88 [69, 96] (21/24)
SCT^*a*^ (B > 2 mm)	100 [89, 100] (0/30)			96 [80, 100] (23/24)		
SCT^*a*^ (B ≥ 4 mm)	100 [89, 100] (0/30)			96 [80, 100] (23/24)		
CCT^*b*^ (B > A)	97 [83, 100] (1/30)			100 [86, 100] (24/24)		
CCT^*b*^ (B-A > 2 mm)	100 [89, 100] (0/30)			96 [80, 100] (23/24)		
CCT^*b*^ (B-A > 4 mm)	100 [89, 100] (0/30)			79 [60, 91] (19/24)		

^*a*^ SCT, single cervical tuberculin skin test (skin reaction increases for PPD-B at different cut-offs).

^*b*^ CCT, comparative cervical tuberculin skin test (skin reaction increases for PPD-B minus PPD-A [B-A] at different cut-offs).

As in previous experiments, the IGRA was applied to stimulated blood samples from both experimental groups. There was no significant difference in the magnitude of the IGRA response in experimentally infected animals for either MDT-F13.1 and MDT-F when compared to MDT-F13 (*P* > 0.05, Friedman test with Dunn’s multiple comparison test; Fig. S4). Additionally, the magnitude of the IGRA response to any of the MDT formulations did not significantly differ from that observed for the B-A readout (*P* > 0.05, Friedman test with Dunn’s multiple comparison test). In the control animals, all the MDT formulations induced very limited (if at all) IGRA responses. All three MDT formulations and the B-A response gave positive results in all experimentally infected animals (100% sensitivity, 95% CI 86%–100%, *n* = 24). In the control animals, two individuals tested positive with the B-A response (93% specificity, 95% CI 79%–99%, *n* = 30) and one animal tested positive with the MDT-F formulation (97% specificity, 95% CI 83%–100%, *n* = 30); no animals tested positive with MDT-F13 and -F13.1.

## DISCUSSION

Tuberculin skin testing, alongside more recent ancillary blood tests, is the mainstay of numerous surveillance programs that have led to the eradication of BTB in countries such as Australia and many member states of the European Union (2,3). The PPDs, therefore, have played a pivotal role in controlling and eradicating BTB in many countries. However, these reagents, which were developed by Seibert in the 1930s, are not without their limitations, as outlined earlier. Of particular concern at present is that PPD potencies must be determined in an animal model, using international standards in short supply and of waning quality, which could negatively impact the maintenance of PPD quality worldwide. To overcome these limitations, we are developing the MDT formulation, a reagent composed of defined protein antigens that could eventually replace PPD-A and PPD-B. Here, we describe our successful approach to simplifying the MDT formulation with the generation of fusion proteins and the successful expression of a re-designed Rv3616c protein.

The MDT formulation builds on the so-called DIVA Skin Test (DST) reagent currently being evaluated as a single fusion protein DST-F in field trials in Great Britain (15,16). While the DST-F is composed of ESAT6, CFP10, and Rv3615c (17), the MDT contains five additional antigens (14), including another ESAT6 family member, Rv3020c (18), and Rv3616c (EspA), another component of the esx-1 secretion system. Unlike the DST, the MDT has no DIVA functionality, as it was not designed to be used in BCG-vaccinated cattle, and therefore, the formulation can benefit from a wider repertoire of antigens (14).

As in the case of the transition from a multi-protein DST-C formulation to a single fusion protein DST-F, it is hoped that simplification of the MDT formulation will yield similar advantages in terms of ease of manufacture and the associated quality control processes (19). As with the DST-F reagent, the generation of fusion proteins to cover the individual antigens proved to be largely successful and led to the development of the MDT-F20. Due to the relatively small sizes (around 100 amino acids each) of ESAT6, CFP10, Rv3615c, and Rv3020c, it was possible to present them in a single fusion protein. However, the other proteins are considerably larger, and therefore, the fusion proteins were limited to two antigens each. At this stage, despite the failure to produce an Rv3616c fusion protein, the construction of three fusion proteins successfully reduced the formulation (MDT-F20), and this proved to be as potent a skin and IGRA test reagent as the original formulation comprising individual recombinant proteins (Fig. 1; Fig. S1). This mirrored earlier findings with the DST, where individual proteins and their fusion protein counterpart (DST-F) performed equally well (19).

The issues encountered in the production of an Rv3616c fusion protein were not entirely unexpected, as previous efforts to produce a single recombinant protein had also failed (14). The strategy presented here aimed to eliminate the protein’s lytic properties by structurally altering it with the removal of its non-antigenic regions. This differs from conventional approaches, which aim to remove the lytic elements but retain other biological functions that could then be studied using such a modified protein. Hence, the focus of this work was to retain Rv3616c’s antigenicity and not its inherent biological function(s). When the molarity of the novel Rv3616c-S4 protein and the peptide presentations was taken into account (Fig. 4), the novel S4 demonstrated comparable antigenicity to the peptide pool. The inclusion of S4 in the MDT-S4 skin test formulation did not result in a change in the magnitude of the skin test formulation, relative to formulations containing the Rv3616c peptide pool (Fig. 5). The authors note that if the IFN-γ responses were to be compared using concentration, then the peptide pool would appear to be significantly more immunogenic *in vitro*. This was also seen in a previous study comparing the peptide and protein equivalents of the DST (16), but as observed here, this did not translate into a significant difference *in vivo* in the skin test. With regard to the Rv3616c peptide pools compared in Fig. 5, this experiment also demonstrated that it was possible to reduce the peptide pool from 22 to 13 peptides (MDT-F13; Fig. 5; Fig. S3) without loss of signal strength or non-specific reactions, thereby making the MDT-F13 a viable option should it have not been possible to produce a fusion protein containing Rv3616c.

With the production of the novel Rv3616c-S4 protein, it would have been ideal to be able to evaluate every possible orientation of the two fusion proteins manufactured by Lionex (Rv1789_Rv3616c-S4 and Rv3616c-S4_Rv3478) *in vitro*. In addition to composition, the orientation of the fused proteins can also affect antigenicity as changes to folding and structural conformation can (i) expose or mask epitopes and (ii) affect how the fusion protein is internalized and processed by antigen-presenting cells, potentially altering the pool of major histocompatibility complex-presented epitopes. Nonetheless, we were still able to formulate and test a full fusion MDT formulation (MDT-F) that did not carry a duplicated Rv3810.

The MDT-F performed well in the skin testing of experimentally infected animals, showing comparable signal strength to the CCT (B-A; Fig. 6) and sufficient signal strength to allow for more stringent cut-offs if required (>3 mm and >4 mm; Table 2). Should the MDT-F’s performance be replicated under field conditions, the application of more stringent cut-offs could be of use in the case of export certification or trade assurance and advantageous for surveillance in low-prevalence areas (increasing the positive predictive value of the test). In non-infected animals, the MDT-F showed a small non-specific response (1 mm) in one animal, which could be compensated for by adjusting the cut-offs; at CCT cutoffs relevant to the GB national testing program (>2 mm and >4 mm), the MDT-F performed as well as the CCT. In the MDT-F13.1 formulation, the duplicated Rv3810 was removed, leaving Rv1789 as a stand-alone protein, while retaining Rv3616c as a 13-peptide pool (Table 1). Given the slight drop in signal strength in experimentally infected animals observed with MDT-F13.1 relative to PPD-B (Fig. 6) and the more rapid drop in relative sensitivity with increased cut-off (Table 2), there appears to be no advantage in the use of MDT-F13.1 over MDT-13 or MDT-F, in terms of test performance. Lastly, the data presented here (Fig. S4) support the utility of the MDT-F as an IGRA test reagent, but further work will be required to optimize antigen concentration and test cut-off values using naturally infected cattle.

In conclusion, this study significantly advanced our efforts to produce a molecularly defined antigen reagent to replace the PPDs. Future work will need to focus on establishing the production of the fusion formulation MDT-F at Lionex and its evaluation in naturally infected cattle. These are important next steps on the developmental pathway to a registered and marketable product. More generally, our approach to producing variant proteins containing only the antigenic fractions could be useful for other applications.

## MATERIALS AND METHODS

### Animals

#### *In vivo* skin testing round 1

To provide a source of cattle (*Bos taurus taurus*) for *in vivo* skin testing, male calves (5–7 months old, Holstein-Friesians breed or crosses thereof) were obtained from herds in the BTB Low Risk Area of England that were officially TB free for over 5 years (Fig. 1). The animals were then used to form two groups: (i) experimentally *M. bovis* infected calves (*n =* 24), consisting of animals aged 6–7 months experimentally infected with approx. 5,660 CFU of a field strain of *M. bovis* (AF2122/97) via the endobronchial route (infection was confirmed by post mortem and/or culture analysis); (ii) Gudair vaccinated calves (*n* = 30) that were vaccinated aged 5–6 months with 1 mL of the Gudair vaccine (Virbac Ltd, UK) via the subcutaneous route. The experimentally *M. bovis*-infected calves and the Gudair-vaccinated calves were skin tested 8 weeks post-infection or vaccination, respectively. The same groups of calves were also used to provide blood samples for the *in vitro* whole blood IGRA prior to skin testing, at 8 weeks post-vaccination and 7 weeks post-infection.

#### *In vivo* skin testing round 2

Skin test reagents were evaluated in two groups. The evaluation in experimentally infected animals utilized a group of 24 male Holstein or Holstein-cross calves sourced from Denmark (an officially bovine TB-free country) that were part of an ongoing vaccine/challenge experiment (Fig. 5) (20). These animals were randomly split into two groups of 12 animals, with one group vaccinated with Bacillus Calmette-Guérin vaccine (BCG Danish strain 1331; approx. 0.38 × 10^6^ CFU per dose; AJVaccines, Copenhagen, Denmark), while the other 12 acted as a non-vaccinated control group. Calves were between 45 and 60 days of age at the time of vaccination. After 1 year, all calves were experimentally infected with approx. 7,600 CFU of *M. bovis* (AF2122/97) via the endobronchial route (infection was confirmed by post mortem and/or culture analysis). All calves were skin tested 12 weeks post-infection. The non-infected control animals (*n* = 30, aged 5–6 months at the time of skin testing) were sourced from herds in the BTB Low Risk Area of England, as described above for the first round of skin testing. Both groups of animals were used to provide blood samples for the *in vitro* whole blood IGRA prior to skin testing.

#### *In vivo* skin testing round 3

Male calves were sourced from herds in the BTB Low Risk Area of England, as described for the first round of skin testing, and used to form two groups: (i) calves (*n =* 24) were experimentally infected, aged 4–7 months, with approx. 200 CFU of a field strain of *M. bovis* (AF2122/97) via the endobronchial route (infection was confirmed by post mortem and/or culture analysis); (ii) non-infected control calves (*n* = 30), aged 6–9 months at the time of skin testing. The experimentally infected animals were skin tested 8 weeks post-infection. Both groups of animals were used to provide blood samples for the *in vitro* whole blood IGRA prior to skin testing.

For the analyses undertaken for Fig. 3 and 4, PBMCs were sourced from both experimentally (*n* = 15) and naturally infected cattle (*n* = 17). The experimentally infected animals were bled for PBMCs at 7 weeks post-infection, prior to round 1 of skin testing (see above). Tuberculin skin test positive naturally infected cattle (males and females of four different breeds ranging in age from 8 to 26 months) were sourced from dairy and beef farms in GB with an ongoing BTB breakdown; infection was confirmed by post mortem and/or culture analysis.

### Preparation of skin test formulations

#### *In vivo* skin testing round 1

For the MDT formulation ESAT-6, CFP-10, Rv1789, Rv3020c, Rv3478, Rv3615c, and Rv3810 were sourced as recombinant proteins from a commercial manufacturer (Lionex; Fig. 1). Rv3616c was prepared as a synthetic pool of 20 peptides comprising sixteen 40-mers, three 25-mers, and one 20-mer (GenScript Biotech, Netherlands) where each individual lyophilized peptide was first reconstituted in phosphate-buffered saline (PBS; Life Technologies, UK) or dimethyl sulfoxide (DMSO; Sigma-Aldrich, UK) to a concentration of 10 mg/mL and then combined together to obtain a peptide pool of 0.5 mg of each peptide/mL, diluted in PBS. Details of the composition of the different MDT formulations and the Rv3616c peptide pools, with concentrations, are shown in Table S1. The MDT skin test reagent was then formulated by combining ESAT-6, CFP-10, Rv1789, Rv3020c, Rv3478, Rv3615c, and Rv3810 proteins with the Rv3616c peptide pool so that each protein or individual peptide was at a concentration of 100 µg/mL. For the MDT-F20 formulation, the three fusion proteins (Lionex), namely ESAT6_CFP10_Rv3615c_ Rv3020c (400 µg/mL); Rv1789_Rv3810 (200 µg/mL); and Rv3478_Rv3810 (200 µg/mL) were combined with the Rv3616c peptide pool described above.

#### *In vivo* skin testing round 2

For the formulation of the MDT skin test reagents utilized in the second round of skin testing, namely MDT-F22, MDT-F13, and MDT-S4, a second batch of peptides was procured for Rv3616c (GenScript Biotech) in which one peptide had to be replaced with a set of three overlapping peptides (see Table S1), taking the total number of peptides from 20 to 22 (Fig. 5). The lyophilized Rv3616c peptides were first reconstituted in PBS or DMSO. The solubilization of individual peptides in PBS was optimized as required by the addition of a maximum of 3.0% ammonia water or 9% formic acid. Concentrations of the Rv3616c peptides upon reconstitution were 20 mg/mL, 30 mg/mL, or 40 mg/mL. Individual peptides were then combined as required to obtain two pools of either 22 or 13 peptides, with 0.5 mg of each peptide/mL. In this round of skin testing, the MDT-F22 and MDT-F13 formulations once again combined the fusion proteins with the requisite Rv3616c peptide pool, while in MDT-S4, the peptide pool was replaced with the novel Rv3616c-S4 (Tables 1 and S1; Lionex).

#### *In vivo* skin testing round 3

The formulations were prepared as per the MDT-F13 utilized in the second round of skin testing with the following modifications (Fig. 6): (i) in MDT-F13.1, the Rv1789_Rv3810 was replaced with Rv1789 (Lionex) at 100 µg/mL, and (ii) in MDT-F, the Rv3616c peptide pool was not included, and Rv1789_Rv3810 was replaced by Rv1789_Rv3616c-S4 (Lionex) at 200 µg/mL.

For all skin tests, the MDT formulations were stored at −80°C until required, and purified protein derivatives from *M. avium* (PPD-A; 25,000 IU/mL) and *M. bovis* (PPD-B; 30,000 IU/mL) were obtained from a commercial manufacturer (Prionics, Switzerland).

### Skin test procedure

Injection sites located at the border of the anterior and middle third of the neck on either side of the cow were clipped, and skin thickness was recorded. PPD-A and PPDB were administered in a 0.1 mL volume via intradermal injection as per the manufacturer’s recommendations. MDT reagents were administered in the same way so that a single protein or peptide was delivered at a 10 µg dose in the 0.1 mL volume; the tetrafusion protein was delivered at 40 µg, and the two protein fusion proteins at a 20 µg dose in the 0.1 mL volume. To account for potential injection site differences, a Latin Square design was applied with animals randomly assigned to the Latin Square combinations; the operators were blinded to the nature of the injection solutions. After 72 hours, the skin test sites were checked for reactions by palpation of the skin. If palpable reactions were detected, the skin thickness at the injection site was re-measured and recorded. If no palpable reaction was detected, the pre-injection values were used. Results are expressed as the increase in skin thickness at 72 hours compared to the thickness pre-injection (Δ skin thickness in mm).

### Antigens for *in vitro* assays

For the *in vitro* stimulations, each of the MDT skin test formulations was diluted to yield 0.1 µg/mL for each peptide or individual protein, 0.4 µg/mL for the tetrafusion protein, and 0.2 µg/mL for the double fusion protein. Complete medium (14) was utilized as a diluent for PBMC stimulations and RPMI-1640 medium (Gibco, UK) for the whole blood stimulations. The 22-peptide pool for Rv3616c (Table S1) was prepared from the reconstituted stock preparations described above. The 48 overlapping Rv3616c peptides (Table S2; GenScript Biotech) were supplied lyophilized and reconstituted at 10 mg/mL or 20 mg/mL in either PBS (with 0.5% or 2.0% ammonia water) or DMSO. The Rv3616c-S4 protein, supplied solubilized (Lionex), was diluted in complete medium for a starting concentration of 10 µg/mL. All test antigens were stored at −80°C in 96-well plates until required.

### *In vitro* stimulation of whole blood

Heparinized blood samples were stored overnight at room temperature before stimulation with and without antigens at a final volume of 275 µL/well in duplicate wells of a 96-well plate for 20–24 h at 37°C in 5% CO_2_. As positive controls, blood samples were cultured with pokeweed mitogen (PWM, 10 µg/mL; Sigma, UK), PPD-B, and PPD-A (at 300 IU/mL and 250 IU/mL, respectively). RPMI-1640 medium alone (Gibco, UK) was utilized as a negative control. After stimulation, blood was centrifuged at 300 g for 15 min, and the plasma supernatant was harvested and stored at −80°C until required.

### *In vitro* stimulation of PBMCs

PBMCs were isolated from heparinized cattle blood by density gradient centrifugation using Histopaque 1077 (Sigma, UK) and cryopreserved in fetal calf serum (FCS; Sigma, UK) containing 10% DMSO (Sigma, UK) prior to use. Cryopreserved PBMCs were thawed as quickly as possible in a water bath at 37°C before adding complete medium. After centrifugation at 350 g for 10 min at room temperature, the supernatant was discarded, the cell pellet gently loosened and re-suspended in complete medium, and the cells counted using a hemocytometer. PBMCs were plated at 2 × 10^5^ cells/well in duplicate wells of a 96-well plate and stimulated with and without antigens at a final volume of 275 µL/well for 3 days at 37°C in the presence of 5% CO_2_. The Rv3616c 22 peptide pool and the novel Rv3616c-S4 protein were tested at 10 concentrations for each peptide/protein, between 10 µg/mL and 0.0003 µg/mL, inclusive. Pokeweed mitogen (10 µg/mL; Sigma, UK), PPD-B, and PPD-A (at 300 IU/mL and 250 IU/mL, respectively) were utilized as controls. Complete medium alone was utilized as a negative control. Following incubation, the cell supernatants were removed and stored at -80°C until required.

### IFN-γ release assay

IFN-γ in plasma and PBMC culture supernatants was quantified using the commercially available BOVIGAM enzyme-linked immunosorbent assay kit (Thermo Fisher Scientific, UK). Results were expressed as the optical density at 450 nm (OD_450_) for cultures stimulated with antigen minus the OD_450_ for cultures without antigen (i.e., ΔOD_450_). The IGRA data were interpreted using the cut-off for positivity used in routine GB IGRA surveillance, i.e., antigen minus nil-antigen IFN-γ OD_450_ value of >0.1.

### ELISpot assay

PBMCs were isolated from heparinized cattle blood by centrifugation with Histopaque-1077 (Sigma-Aldrich, UK) and cryopreserved in FCS containing 10% DMSO (Sigma-Aldrich, UK) and stored frozen. The ELISpot assay was essentially carried out as described by Steinbach et al. (21) with the modifications described herein. Following wetting and washing, the 96-well MultiScreen_HTS_ IP filter plates (Merck, Millipore, UK) were coated with 100 µL/well of 7.5 µg/mL of anti-bovine IFN-γ (monoclonal antibody MT17.1 Mabtech, Sweden). Coated plates were sealed and stored at 4°C–8°C overnight or for a maximum of a week prior to washing and blocking. All antigen stocks were diluted to 20 µg/mL in complete media with 0.2% DMSO and tested in duplicate wells at 100 µL/well. The control antigens, PWM, PPD-B, and PPD-A, were utilized as described by Steinbach et al. (21). Negative control wells contained complete medium with 0.2% DMSO. Each well was incubated with 2 × 10^5^ PBMCs in a final volume of 200 µL per well for 20–24 h. The plates were then washed and incubated with 100 µL/well of a biotinylated monoclonal anti-bovine IFN-γ detection antibody (MT307-BAM, 0.25 µg/mL; Mabtech, Sweden) diluted in 0.5% FCS/PBS. After washing, the plates were then incubated for 1 h at room temperature with 100 µL/well Streptavidin-HRP (Mabtech, Sweden) diluted 1:100 in 0.5% FCS/PBS. After washing, the plates were incubated in the dark for 7–8 min with 100 µL/well of AEC prepared according to the manufacturer’s instructions (AEC101 Staining Kit, Sigma, UK). Developed plates were washed with distilled water and air-dried in the dark. IFN-γ-producing cells were counted as spot-forming cells using an ELISpot reader (AID, Germany).

### Statistical analyses

All statistical analyses were performed using GraphPad Prism (GraphPad Software, Version 10.2.3, USA). Comparisons between the skin test responses or the whole blood IGRA responses induced with the antigens were analyzed using the Friedman test with Dunn’s multiple comparisons test; the relevant statistical details are only included in the Figure legend where significant differences were observed. For the ELISpot data, the mean spot forming count (SFC) per peptide/animal was only utilized in the analyses if both SFC values from the duplicate wells were in agreement, i.e., either both below or both above a 95% CI cut-off. The 95% CI cut-off was calculated as: mean NIL SFC + (SEM NIL SFC * *t* value) with the *t* value being the critical value of *t* for 95% CI for a specific degree of freedom (DF) (22). The DF was calculated as either the total number of NIL wells across all the 96-well plates used to test all 48 peptides for an individual animal (DF = *n* NIL – 1), or if the NIL SFCs differed significantly across the plates (Mann-Whitney test), then separate cutoffs and response levels were calculated for each plate with the DF calculated from the number of NIL wells on each plate. The three levels of response to a peptide were calculated as follows: (i) low: >95% CI cut-off and ≤2× mean NIL SFC; (ii) medium: >2× mean NIL SFC and ≤3× mean NIL SFC; and (iii) high: >3× mean NIL SFC. Individual peptides were identified for removal on the basis of (i) the number of animals showing an immunological response, (ii) the strength of the response (low, medium, or high responders), and (iii) proximity to highly antigenic regions, i.e., non- or hypo-antigenic peptides occurring adjacent to highly antigenic peptides were not removed due to the overlap between the peptides. For the *in vitro* comparison of the Rv3616c novel S4 and the Rv3616c peptide pool (Fig. 4), the molarity (nM) of each formulation for a given concentration was calculated using the molecular weight of the amino acid sequence of each peptide and the protein. For the peptide pool, the overall molarity of the cocktail was calculated by summing the number of moles of each peptide at a given concentration. The Wilcoxon matched-pairs signed rank test was used to compare the ΔOD_450_ responses of Rv3616c-S4 and the 22 peptide pool for five comparable molarities (inset panel in Fig. 4) between 2.02 nM and 287 nM.

## Data Availability

The data sets used and/or analyzed during the current study are available from the corresponding author on reasonable request.

## SUPPLEMENTAL MATERIAL

Supplemental Material jcm.00552-25-s0001.pdf Figures S1 to S4; Tables S1 and S2.

ASM does not own the copyrights to Supplemental Material that may be linked to, or accessed through, an article. The authors have granted ASM a non-exclusive, world-wide license to publish the Supplemental Material files. Please contact the corresponding author directly for reuse.
