# Supplementary material for: Optimization of a molecularly defined tuberculin formulation: recombinant fusion proteins and epitope surgery
Source: J Clin Microbiol. 2025 Aug 29;63(10):e00552-25. doi: 10.1128/jcm.00552-25 (PMC12505890; doi:10.1128/jcm.00552-25)
Supplement: Supplemental Material — Figures S1 to S4; Tables S1 and S2. [file jcm.00552-25-s0001.pdf]

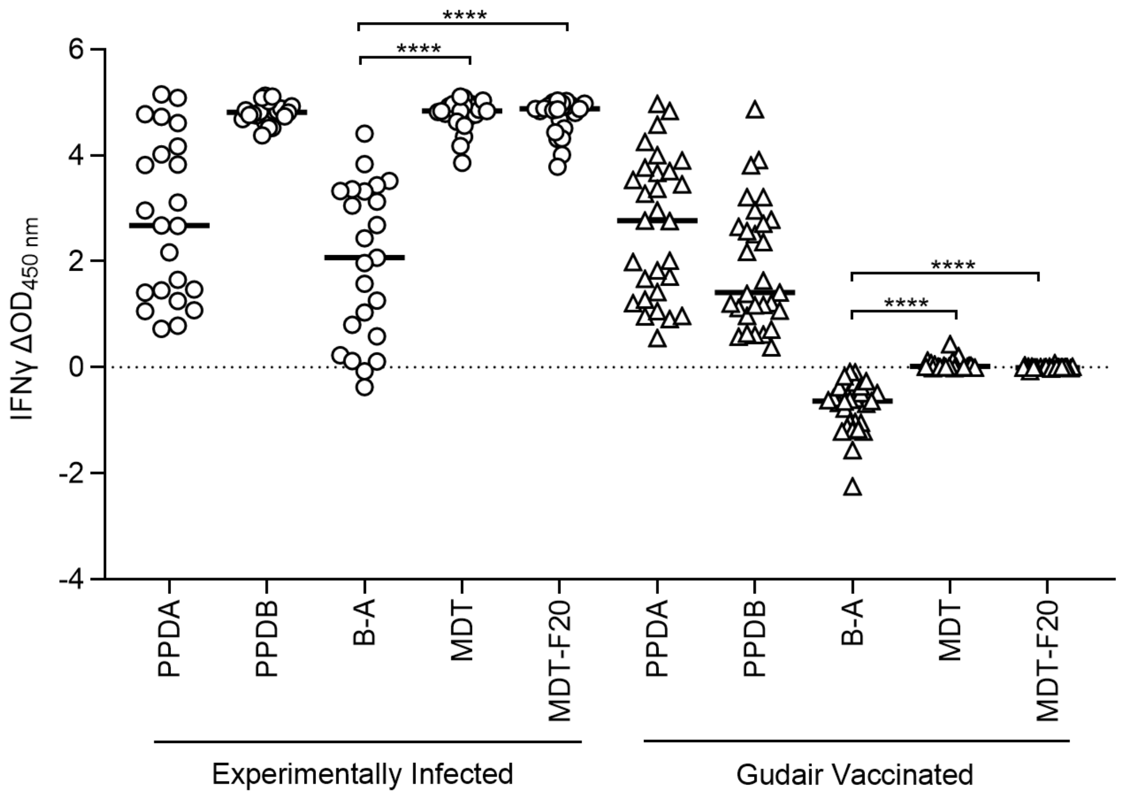

Figure S1. Quantification of MDT and MDT-F20 induced *in vitro* IFN-γ production. Blood samples from cattle experimentally infected with *M. bovis* ($n = 23$) and Gudair vaccinated cattle ($n = 29$) were stimulated with PPDs, MDT and MDT-F20 reagents and IFN-γ production measured by ELISA. Each symbol represents an individual animal while horizontal lines represent group medians. **** $p < 0.0001$, Friedman test with Dunn's multiple comparisons test.

MSRAFIIDPTISAIDGLYDLLGIGIPNQGGILYSSLEYFEKALEELAAAFPGDGWLG
SAADKYAGKNRNHVNFFQELADLDRQLISLIHDQANAVQTTRDILEGAKKGLEFV
RPVAVDLTYIPVVGHALSAAFQAPFCAGAMAVVGGALAYLVVKTLINATQLLKLL
AKLAELVAAAIADIISDVADIIKGTLGEVWEFITNALNGLKELWDKLTGWVTGLFS
RGWSNLESFFAGVPGLTGATSGLSQVTGLFGAAGLSASSGLAHADSLASSASLPA
LAGIGGGSGFGGLPSLAQVHAASTRQALRPRADGPVGAAAEQVGGQSQLVSAQ
GSQGMGGPVGMGGMHPSSGASKGTTTKKYSEGAAAGTEDAERAPVEADAGG
GQKVLVRNVV

Figure S2. The wild-type amino acid sequence of Rv3616c (*M. bovis* AF2122/97) with the three deleted regions highlighted in grey. A total of 50 of the 392 residues were deleted across three regions of 28, 12 and 10 residues.

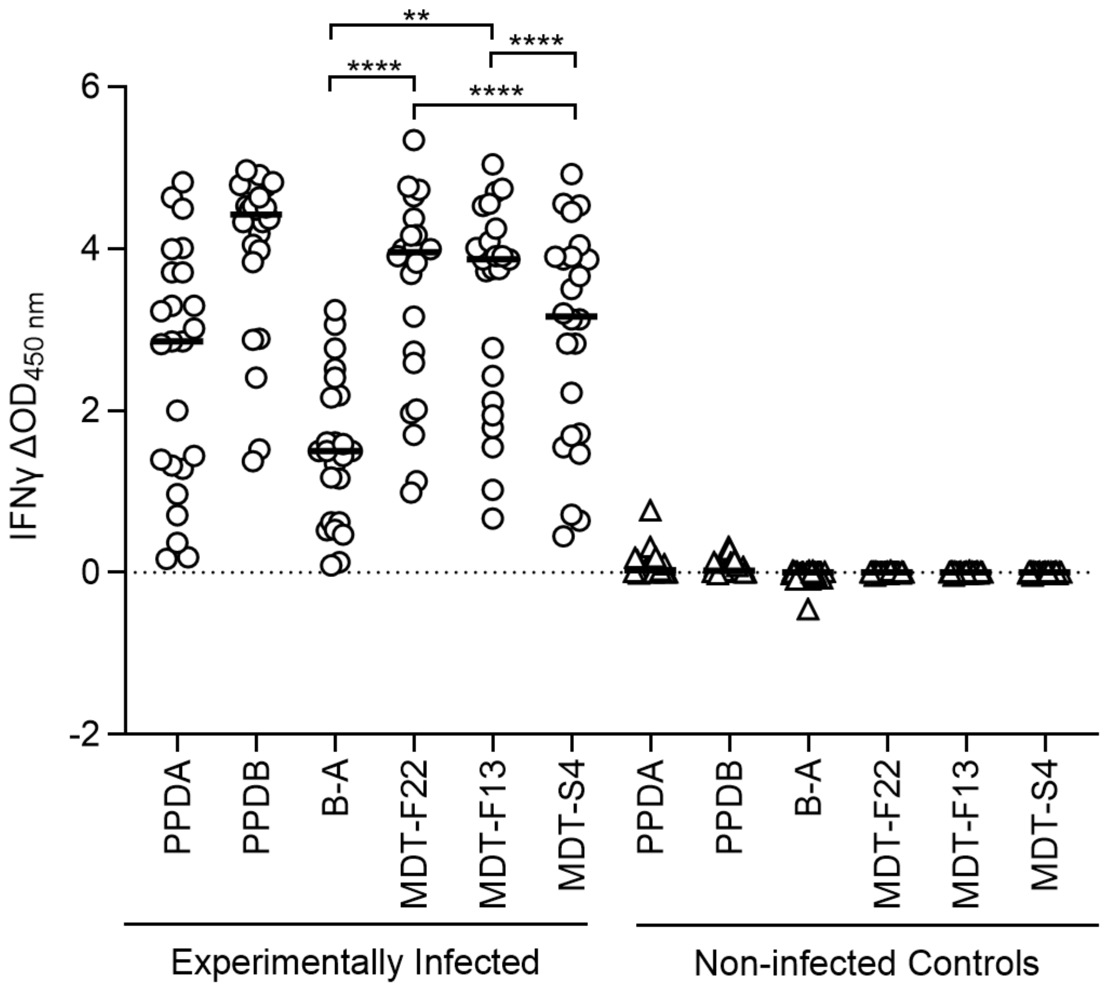

Figure S3. Quantification of MDT-F22, MDT-F13 and MDT-S4 induced *in vitro* IFN-γ production. Blood samples from cattle experimentally infected with *M. bovis* ($n = 24$) and non-infected controls ($n = 30$) were stimulated with PPDs, MDT-F22, MDT-F13 and MDT-S4 reagents and IFN-γ production measured by ELISA. Each symbol represents an individual animal while horizontal lines represent group medians. **** $p < 0.0001$, ** $p = 0.001$, Friedman test with Dunn's multiple comparisons test.

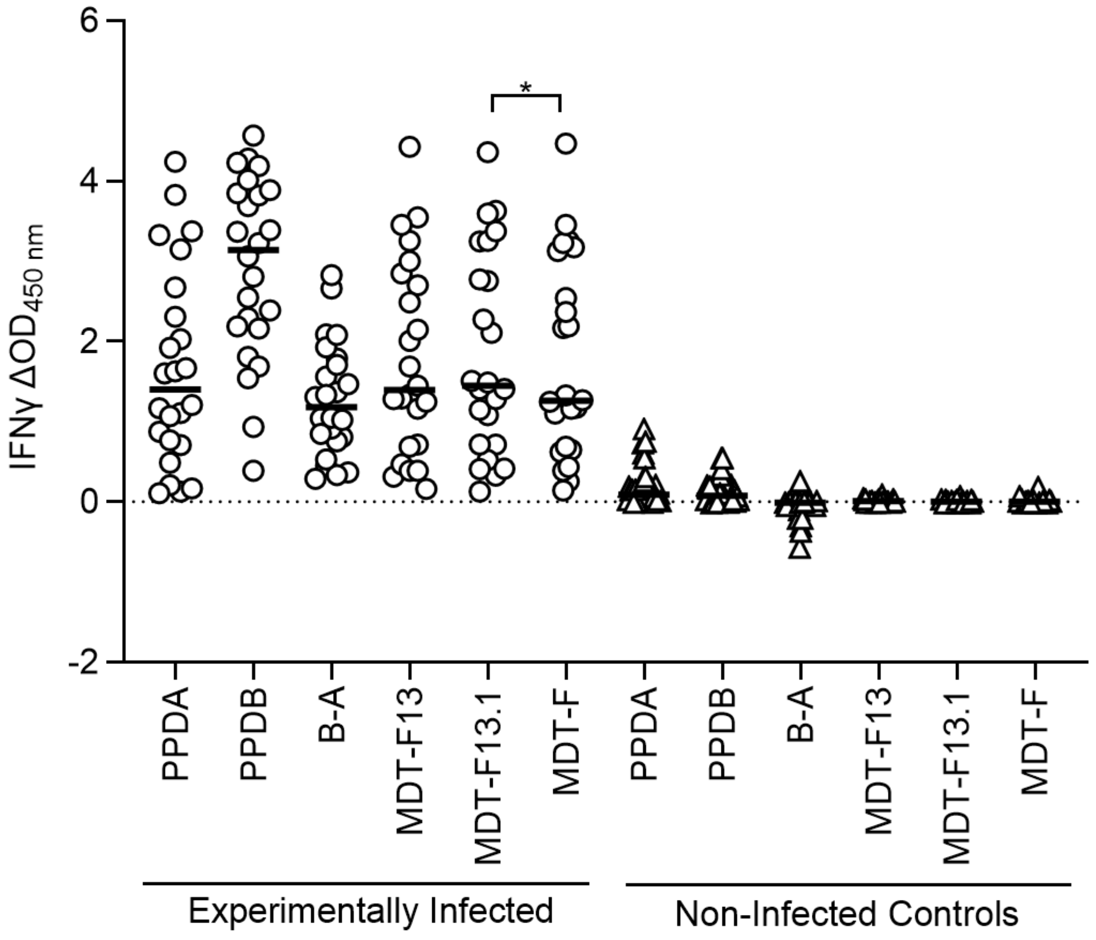

Figure S4. Quantification of MDT-F13, MDT-F13.1 and MDT-F induced *in vitro* IFN-γ production. Blood samples from cattle experimentally infected with *M. bovis* (n = 24) and non-infected controls (n = 30) were stimulated with PPDs, MDT-F13, MDT-F13.1 and MDT-F reagents and IFN-γ production measured by ELISA. Each symbol represents an individual animal while horizontal lines represent group medians. * $p = 0.018$, Friedman test with Dunn's multiple comparisons test.

Table S1. Individual components of the MDT formulations and their concentrations (µg/ml) in the skin test formulations. X – not included in the formulation. *The Rv3616c peptide pool in the MDT-F20 formulation increased from 20 to 22 peptides (MDT-F22) when Rv3616c Peptide 10 could no longer be manufactured and had to be replaced with 3 x 24-mer peptides with a 12 amino acid overlap (Rv3616c-10_1/2/3).

Components		MDT Formulations (µg/ml/protein or peptide)						
		MDT	MDT-F20	MDT-F22*	MDT-F13	MDT-S4	MDT-F13.1	MDT-F
Individual recombinant proteins	ESAT6	100	X	X	X	X	X	X
	CFP10	100	X	X	X	X	X	X
	Rv3615c	100	X	X	X	X	X	X
	Rv3020c	100	X	X	X	X	X	X
	Rv1789	100	X	X	X	X	100	X
	Rv3810	100	X	X	X	X	X	X
	Rv3478	100	X	X	X	X	X	X
	Rv3616c-S4	X	X	X	X	100	X	X
Fusion Proteins	ESAT6-CFP10-Rv3615c-Rv3020c	X	400	400	400	400	400	400
	Rv1789-Rv3810	X	200	200	200	200	X	X

Components		MDT Formulations (μg/ml/protein or peptide)						
		MDT	MDT-F20	MDT-F22*	MDT-F13	MDT-S4	MDT-F13.1	MDT-F
	Rv3478-Rv3810	X	200	200	200	200	200	200
	Rv1789-Rv3616c-S4	X	X	X	X	X	X	200
Rv3616c Peptides	Rv3616c-1	100	100	100	100	X	100	X
	Rv3616c-2	100	100	100	X	X	X	X
	Rv3616c-3	100	100	100	X	X	X	X
	Rv3616c-4	100	100	100	100	X	100	X
	Rv3616c-5	100	100	100	X	X	X	X
	Rv3616c-6	100	100	100	X	X	X	X
	Rv3616c-7	100	100	100	X	X	X	X
	Rv3616c-8	100	100	100	100	X	100	X
	Rv3616c-9	100	100	100	X	X	X	X
	Rv3616c-10	100	100	X	X	X	X	X
	Rv3616c-11	100	100	100	100	X	100	X
	Rv3616c-12	100	100	100	100	X	100	X

Components		MDT Formulations (µg/ml/protein or peptide)						
		MDT	MDT-F20	MDT-F22*	MDT-F13	MDT-S4	MDT-F13.1	MDT-F
	Rv3616c-13	100	100	100	X	X	X	X
	Rv3616c-14	100	100	100	100	X	100	X
	Rv3616c-15	100	100	100	100	X	100	X
	Rv3616c-16	100	100	100	100	X	100	X
	Rv3616c-17	100	100	100	100	X	100	X
	Rv3616c-18	100	100	100	X	X	X	X
	Rv3616c-19	100	100	100	100	X	100	X
	Rv3616c-20	100	100	100	100	X	100	X
*Replacement peptides for Rv3616c-10	Rv3616c-10_1	X	X	100	100	X	100	X
	Rv3616c-10_2	X	X	100	X	X	X	X
	Rv3616c-10_3	X	X	100	100	X	100	X

Table S2. The 48 overlapping peptide set spanning the length of the Rv3616c native protein, utilised to identify the non- and hypo-antigenic regions for the purpose of designing a novel Rv3616c protein.

Peptide Number	Length (mer)	AA Sequence
1	20	MSRAFIIDPTISAIDGLYDL
2	20	PTISAIDGLYDLLGIGIPNQ
3	20	LYDLLGIGIPNQGGILYSSL
4	20	IPNQGGILYSSLEYFEKALE
5	20	YSSLEYFEKALEELAAAFPG
6	20	KALEELAAAFPGDGWLGSAA
7	20	AFPGDGWLGSAADKYAGKNR
8	20	GSAADKYAGKNRNHVNFFQE
9	20	GKNRNHVNFFQELADLDRQL
10	20	FFQELADLDRQLISLIHDQA
11	20	DRQLISLIHDQANAVQTTRD
12	20	HDQANAVQTTRDILEGAKKG
13	20	TTRDILEGAKKGLEFVRPVA
14	20	AKKGLEFVRPVAVDLTYIPV
15	20	RPVAVDLTYIPVVGHALSAA
16	20	YIPVVGHALSAAFQAPFCAG
17	20	LSAAFQAPFCAGAMAVVGGA
18	20	FCAGAMAVVGGALAYLVVKT
19	20	VGGALAYLVVKTLINATQLL
20	20	VVKTLINATQLLKLLAKLAE
21	20	TQLLKLLAKLAELVAAAIAD
22	20	KLAELVAAAIADIISDVADI
23	20	AIADIISDVADIIKGTLGEV
24	20	VADIIKGTLGEVWEFITNAL
25	20	LGEVWEFITNALNGLKELWD
26	20	TNALNGLKELWDKLTGWVTG
27	20	ELWDKLTGWVTGLFSRGWSN
28	20	WVTGLFSRGWSNLESFFAGV
29	20	GWSNLESFFAGVPGLTGATS
30	20	FAGVPGLTGATSGLSQVTGL
31	20	GATSGLSQVTGLFGAAGLSA
32	20	VTGLFGAAGLSASSGLAHAD
33	20	GLSASSGLAHADSLASSASL
34	20	AHADSLASSASLPALAGIGG
35	20	SASLPALAGIGGGSGFGGLP
36	20	GIGGGSGFGGLPSLAQVHAA
37	20	GGLPSLAQVHAASTRQALRP
38	20	VHAASTRQALRPRADGPVGA
39	20	ALRPRADGPVGAAAEQVGGQ

Peptide Number	Length (mer)	AA Sequence
40	20	PVGAAAEQVGGQSQLVSAQG
41	20	VGGQSQLVSAQGSQGMGGPV
42	20	SAQGSQGMGGPVGMGGMHPS
43	20	GGPVGMGGMHPSSGASKGTT
44	20	MHPSSGASKGTTTKKYSEGA
45	20	KGTTTKKYSEGAAAGTEDAE
46	20	SEGAAAGTEDAERAPVEADA
47	20	EDAERAPVEADAGGGQKVLV
48	16	EADAGGGQKVLVRNVV
